# Large pneumothorax following thoracic and lumbar tumor surgery: Risk factors and management strategies

**DOI:** 10.3389/fsurg.2023.1066841

**Published:** 2023-01-26

**Authors:** Yan Lou, Yunyan Zhang, Zhongzhao Wang, Chenglong Zhao, Zhenxi Li, Quan Huang, Hao Tang, Jianru Xiao

**Affiliations:** ^1^ Department of Orthopedic Oncology, Spine Tumor Center, Changzheng Hospital, Naval Military Medical University, Shanghai China; ^2^ Department of Respiratory and Critical Care Medicine, Changzheng Hospital, Naval Military Medical University, Shanghai China

**Keywords:** pneumothorax, spinal tumor surgery, complication, en bloc resection, chest drainage

## Abstract

**Objective:**

Large pneumothorax is a rare but dangerous complication following thoracic and lumbar tumor surgery. There is little discussion about the features of large pneumothorax following spinal tumor surgery. The purpose of this study was to analyze the characteristics of postoperative pneumothorax, identify factors related to large pneumothorax, and propose a management algorithm for prevention, diagnosis, and treatment.

**Methods:**

Included in this retrospective study were 118 patients who developed pneumothorax after receiving thoracic and lumbar tumor surgery between January 2015 and October 2021. A measurement of lung compression ≥20% on chest CT or x-ray was defined as large pneumothorax, and potential risk factors for large pneumothorax were identified by univariate analysis.

**Results:**

Spinal tumor history and intraoperative blood loss were risk factors for large pneumothorax. The common symptoms of postoperative pneumothorax were chest pain, chest tightness and dyspnea. The mean longest transverse diameter of tumors was 6.63 ± 2.4 cm. En bloc resection was performed in 70 patients, with a mean operation time of 6.9 ± 2.5 h and mean intraoperative blood loss of 1771 ± 1387 ml. The most common pathologies were chondrosarcoma, giant cell tumors of bone, and neurogenic tumors.

**Conclusion:**

During surgery, an artificial dura mater patch and a prolene suture can be used to repair the pleural and lung defects. We recommend chest CT as the preferred method for identifying postoperative pneumothorax. If a patient presents severe dyspnea, a large pneumothorax or concurrent pleural effusion, application of chest drainage is strongly recommended.

## Introduction

Pneumothorax is a relatively uncommon but potentially fatal complication of thoracic and lumbar tumor surgery (1, 2). Postoperative pneumothorax can be easily missed due to the absence of specific symptoms. If not treated in time, it may result in serious implications such as respiratory failure, atrial fibrillation, or even death (1, 3). The reported incidence of pneumothorax following posterior spine surgery ranges from 0.2% to 1.6% (1, 4, 5).

However, most related studies have focused on the pneumothorax associated with scoliosis surgery or thoracic rod removal procedures (3, 6–9), and little attention has been paid to the diagnosis and treatment of pneumothorax after spinal tumor surgery. In scoliosis surgery, pleural rupture is mostly caused by the placement of pedicle screws and the defect is usually small and easily sutured (10). In contrast, pleural defects caused by spinal tumor resection are much larger and more difficult to repair (11). For this reason, the incidence of pneumothorax associated with spinal tumor surgery is higher with more severe symptoms and worse consequences, constituting a formidable clinical challenge.

In this study, we reviewed patients with pneumothorax following thoracic and lumbar tumor surgery from January 2015 to October 2021, and analyzed their clinical characteristics, imaging findings and surgical procedures in an attempt to sum up some useful clues for the clinical diagnosis, treatment and prevention, hoping that the results could help enhance spine surgeons' awareness and vigilance regarding the occurrence of postoperative pneumothorax. To the best of our knowledge, this is the largest cohort of patients with pneumothorax following thoracic and lumbar tumor surgery in a single center.

## Materials and methods

### Patient selection

**Figure 1:**
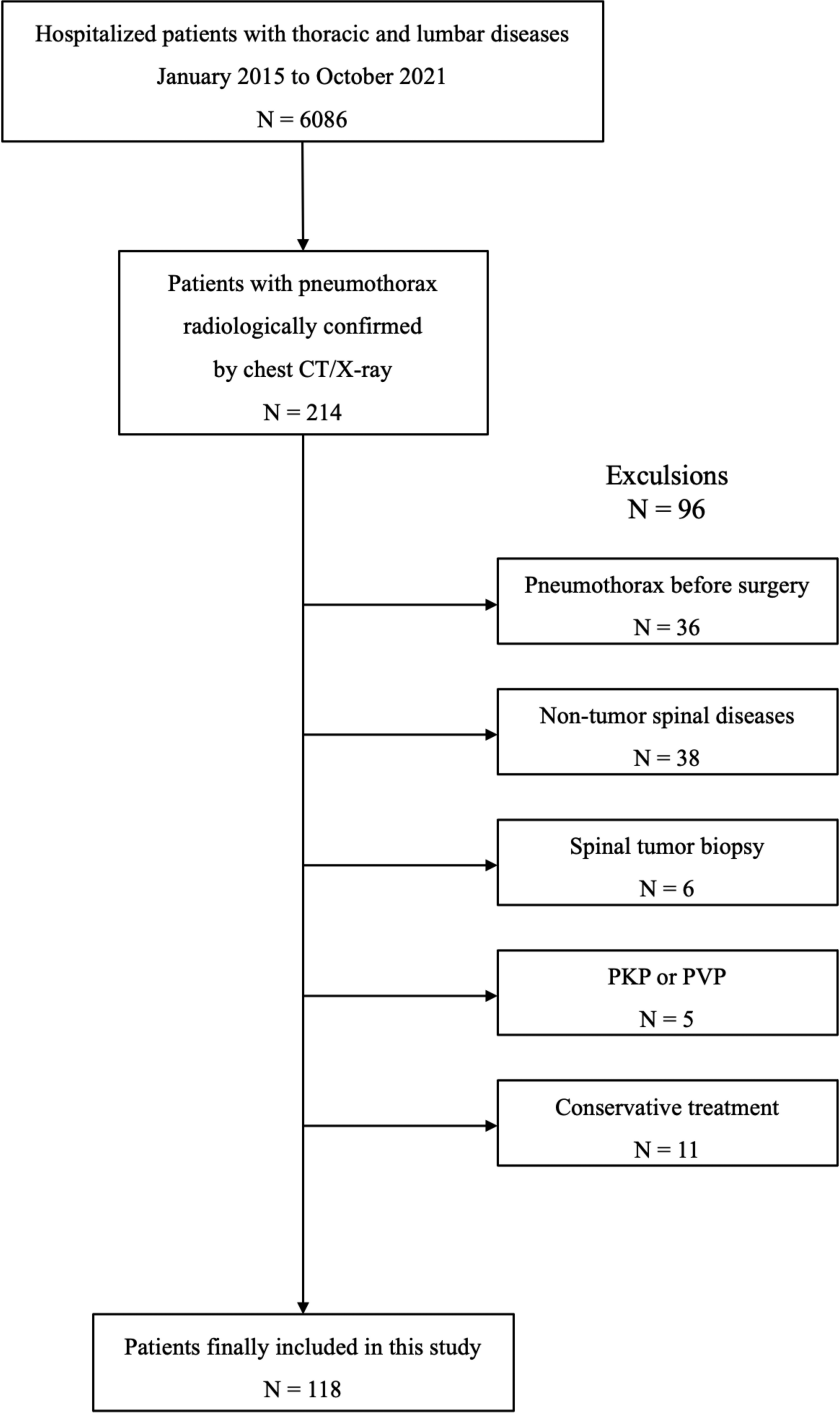
Diagram showing the selection of patients to be included in this study.

We reviewed the electronic medical records and screened out 6,086 hospitalized patients with thoracic and lumbar diseases in our center from January 2015 to October 2021. Based on the chest CT and x-ray reports, 214 patients with pneumothorax during hospitalization were further selected. The inclusion criteria were: (1) patients with primary or metastatic thoracic and lumbar tumors who received surgical treatment; and (2) pneumothorax occurring after surgery whose diagnosis was confirmed by chest CT or x-ray. Exclusion criteria were: (1) patients who had pneumothorax prior to surgery; (2) patients with spinal tumors who underwent puncture or open biopsy; and (3) patients with spinal tumors who underwent percutaneous kyphoplasty (PKP) or percutaneous vertebroplasty (PVP). According to the aforementioned criteria, 118 patients were eventually included in our study (Figure 1). The research protocol was approved by the ethics committee of the said hospital, and written informed consent was obtained from each patient or their legal guardians.

### Study methods

We searched the Picture Archiving and Communication System to measure and calculate the lung compression of the patients with postoperative pneumothorax on chest CT or x-ray, and determine whether these patients had concurrent pleural effusion. The lung compression formula for patients with postoperative pneumothorax of chest CT is 1.159* W/D-1.034 (D is the maximal anteroposterior diameter in the transdiaphragmatic thorax and W is the maximal width of the pneumothorax)(12). The lung compression formula of chest x-ray (CXR) is 4.2 + 4.7*(A + B + C) (A is the interpleural distance of the apex; B is the lateral at the middle of the collapsed lung's upper halves; C is the collapsed lung's lower halves) (13). Lung compression ≥20% was defined as a large pneumothorax (14).

### Statistical analysis

Patients were classified into two groups based on the degree of lung compression: large pneumothorax and small pneumothorax. Categorical data were presented as the frequency (percentage), whereas continuous data were presented as the mean ± standard deviation (SD). Univariate analysis was used to identify potential risk factors for large pneumothorax. The independent *t*-test was used to evaluate continuous variables. The *χ*^2^ test was used to assess categorical data. Fisher's exact test was applied when the theoretical frequency was less than five. Statistical analyses were performed with SPSS (version 27.0, Chicago, Illinois, United States).

## Results

Of the 118 patients enrolled in this study, 62 were male and 56 were female with a mean age of 43.2 ± 17.2 (range 10–79) years. All the pneumothoraces that occurred postoperatively in our series were closed pneumothoraces. The common symptoms were chest pain, chest tightness, and dyspnea. Over two-thirds of the patients (*n *= 79) reported no obvious discomfort. This might be because chest drainage tubes were placed in the surgery in 48 of these patients, and 22 of the remaining 31 asymptomatic patients had small pneumothorax. The tumors were large, with the longest transverse diameter averaging 6.63 ± 2.4 cm. 92.3% (109/118) patients had no history of smoking, and 75.4% (89/118) patients had no underlying lung diseases such as bullae and chronic obstructive pulmonary disease (COPD). The mean body mass index (BMI) of the patients was 22.4, which was within the normal range. Except for two patients who died of non-pneumothorax causes (one died of multiple organ failure and the other of cardiac arrest), the rest of the patients recovered well.

**Figure 2:**
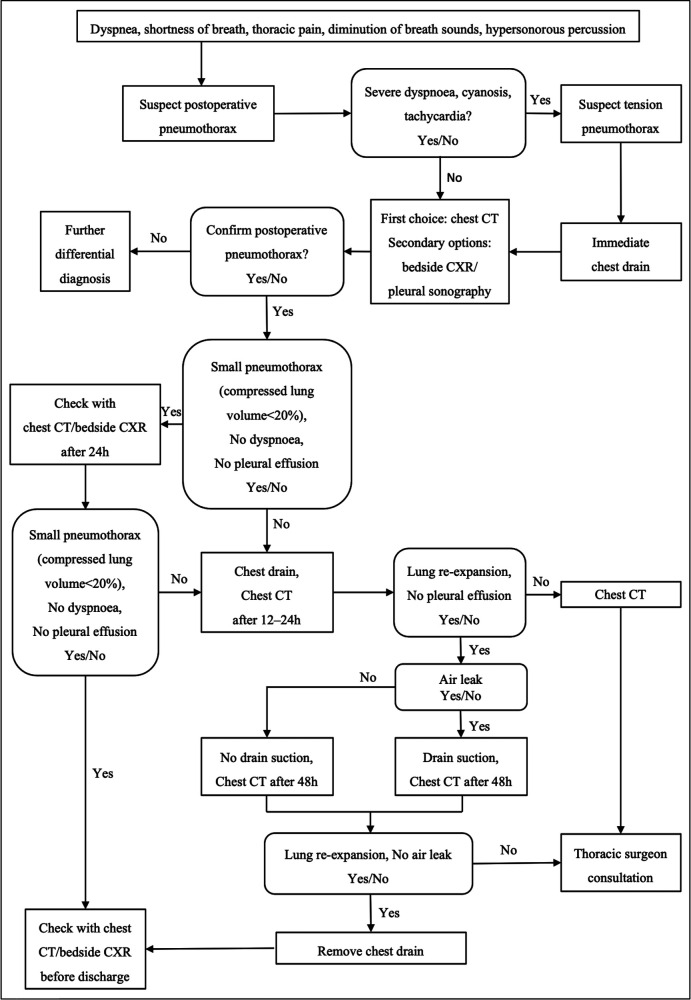
An algorithmic approach to the management of pneumothorax following thoracic or lumbar tumor surgery.

**Figure 3:**
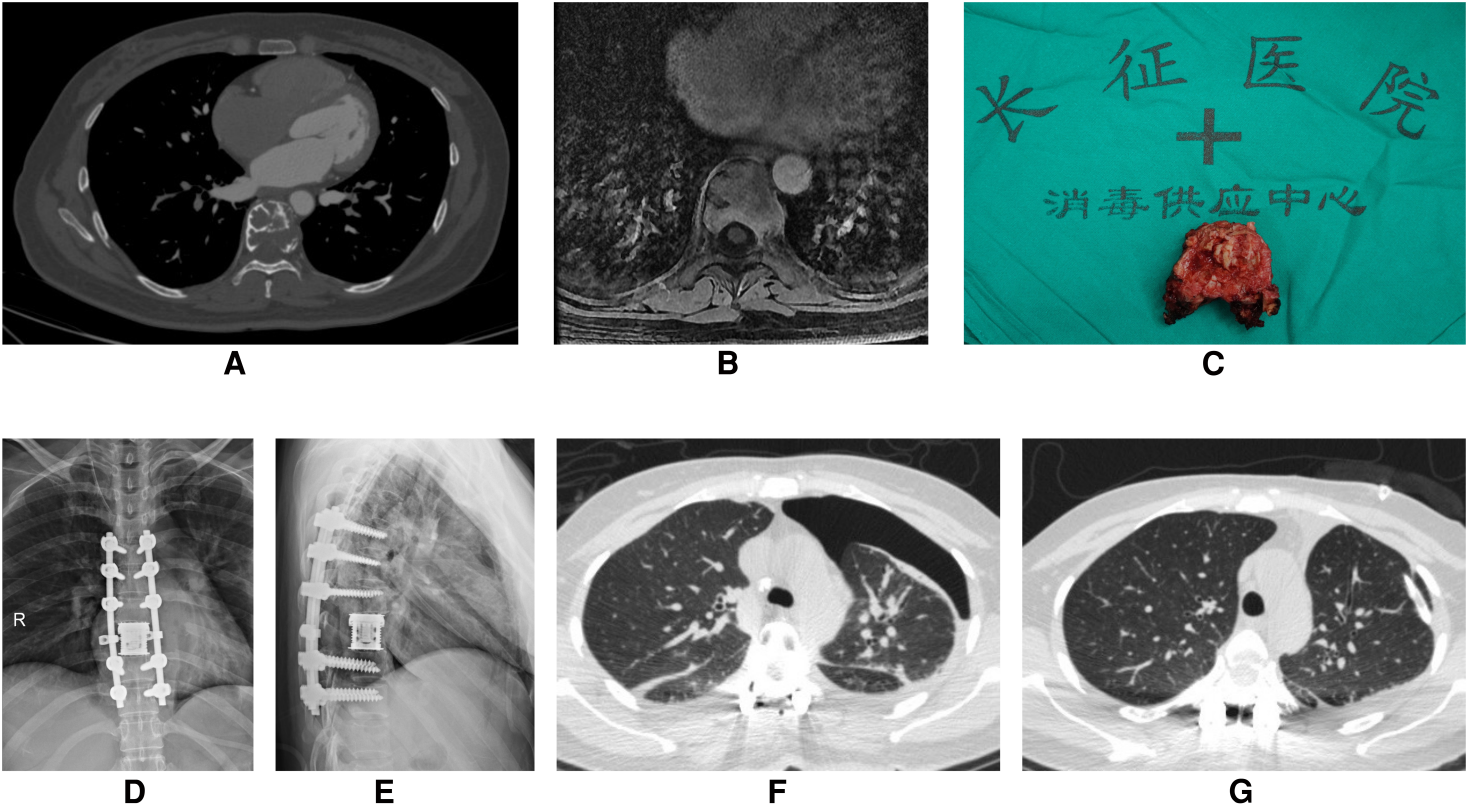
A 43-year-old woman underwent T8 *en bloc* spondylectomy for giant cell tumor of bone through the posterior approach. (**A**) Preoperative enhanced CT showed that T8 vertebral body had osteolytic bone destruction and a soft tissue mass. There were bone ridges and sclerotic rims in the bone destruction area. (**B**) Preoperative enhanced MRI showed bone destruction of T8 vertebral body with heterogeneous enhancement. (**C**) En bloc resection of giant cell tumor of thoracic spine (T8). (**D,E**) Anteroposterior and lateral x-ray of the thoracic spine after surgery. (**F**) On the first postoperative day, Chest CT showed severe left pneumothorax. (**G**) After left chest closed drainage, reexamination of chest CT showed the left lung was re-expanded well.

**Figure 4:**
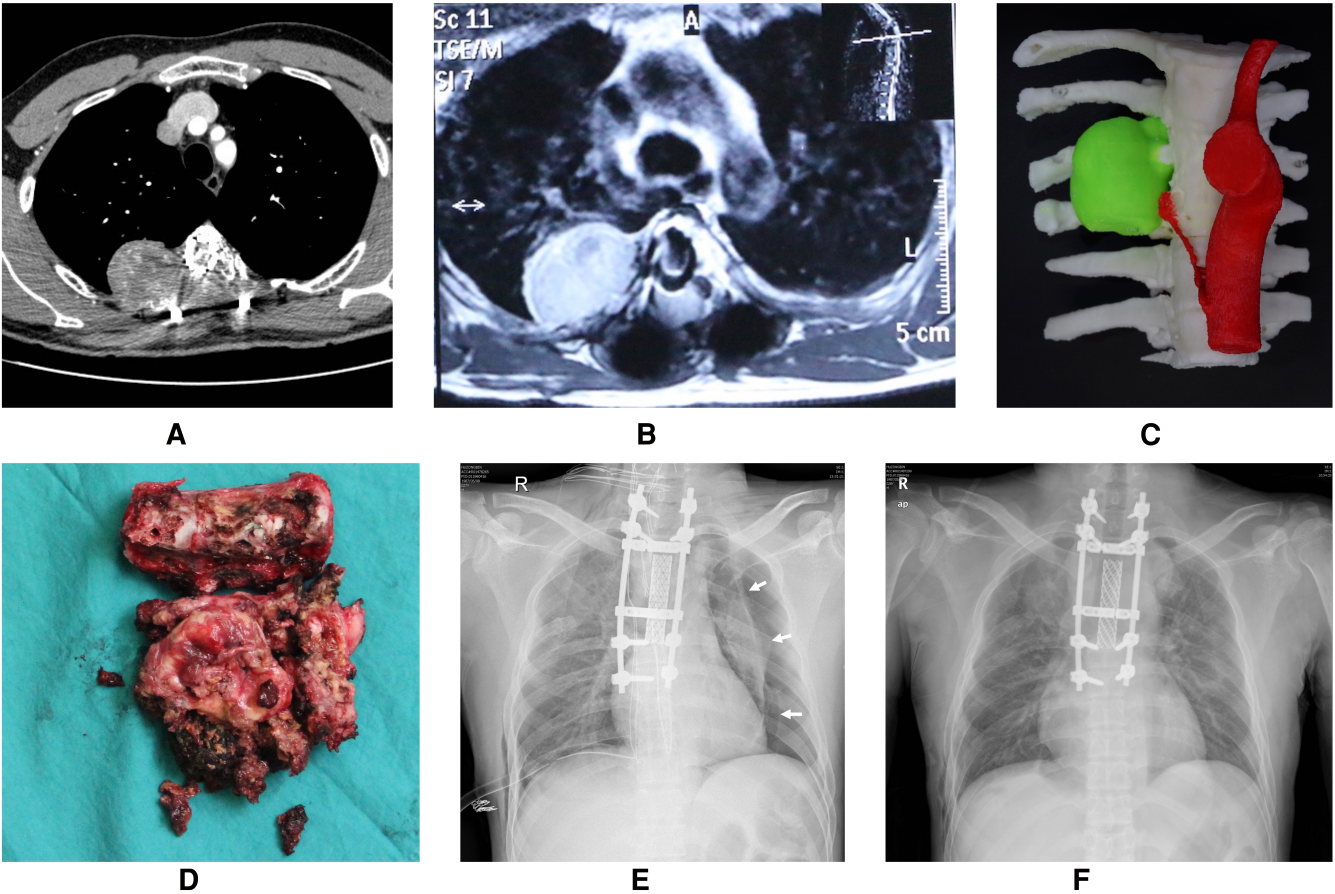
A 28-year-old patient who underwent T3-5 recurrent tumors resection. (**A**) Preoperative CT angiography of the thoracic aorta showed bone destruction in the right part of the vertebral body, lamina, transverse process and corresponding ribs of T3-5, accompanied by the formation of paravertebral soft tissue masses. (**B**) Preoperative enhanced MRI showed oval tumor tissue on the right side of T4. (**C**) 3D-printed model of tumor (green). (**D**) The tumor specimen completely resected in piecemeal. (**E**) Although the right pleura was torn and sutured in the surgery, the bedside chest radiograph indicated a left pneumothorax unexpectedly on the first night after surgery. The white arrow indicate the pneumothorax line. (**F**) The left pneumothorax disappeared, the left lung was re-expanded well, and the bilateral chest drainage tubes were removed at discharge.

Large pneumothorax patients accounted for 72.0% of all postoperative pneumothorax patients. Univariate analysis showed that spinal tumor history and intraoperative blood loss were risk factors for large pneumothorax (Table 1). Based on our analysis and earlier therapeutic experience, we developed an algorithmic strategy for the management of pneumothorax following thoracic and lumbar tumor surgery (Figure 2). We recommend chest CT as the choice of examination to confirm pneumothorax after surgery in patients with stable vital signs. Observation was appropriate for patients with small pneumothorax (lung compression ratio <20%) and without dyspnea and pleural effusion. But a chest CT or bedside CXR should be reviewed after 24 h. If the patient developed progressive dyspnea, a large pneumothorax, or concurrent pleural effusion, application of chest drainage was indicated (Figure 3). For patients whose pleurae were repaired during surgery, chest CT should be reviewed after surgery to exclude pneumothorax and pleural effusion. We had a 28-year-old patient who underwent T3–5 recurrent tumor resection. During the separation of the huge tumor, the right pleura was torn inadvertently and sutured continuously with 4-0 prolene afterwards. After the posterior surgery, a right chest drainage tube was placed in advance to avoid postoperative pulmonary complications. However, on the first night after surgery, pneumothorax occurred on the left side (Figure 4). We considered that although the patient's left pleura was not significantly ruptured in surgery, the residual left pleura was too thin to cause the delayed rupture and left pneumothorax. If closed chest drainage was not satisfactory (e.g., persistent air leakage over 48 h, insufficient re-expansion), thoracic surgery consultation was necessary to consider further treatment.

**Table 1:** Characteristics of patients with pneumothorax following thoracic and lumbar tumor surgery.

Demographics of the patients	Large Pneumothorax [*N* = 85 (72.0%)]	Small Pneumothorax [*N* = 33 (28.0%)]	*p*-value
Age^a^ (year)	43.2 ± 17.3	43.1 ± 17.0	0.961
Sex^b^			0.947
Male	44 (51.8%)	18 (54.5%)	
Female	41 (48.2%)	15 (45.5%)	
BMI^a^ (Kg/m^2^)	22.2 ± 3.5	22.8 ± 3.5	0.399
Smoking history^b^			0.709
Yes	6 (7.1%)	3 (9.1%)	
No	79 (92.9%)	30 (90.9%)	
Underlying lung diseases^b^			0.771
Yes	22 (25.9%)	7 (21.2%)	
No	63 (74.1%)	26 (78.8%)	
Chief complaint^b,c^			0.708
Chest pain/Chest tightness	18 (20.2%)	8 (20.0%)	
Cough/Wheezing	2 (2.2%)	2 (5.0%)	
Severe dyspnea	9 (10.1%)	6 (15.0%)	
Other	3 (3.4%)	2 (5.0%)	
No obvious symptoms	57 (64.0%)	22 (55.0%)	
Bilateral or unilateral pneumothorax^b^			0.620
Unilateral pneumothorax	72 (84.7%)	26 (78.8%)	
Bilateral pneumothorax	13 (15.3%)	7 (21.2%)	
Comorbidity^b^			0.157
Simple pneumothorax	8 (9.4%)	7 (21.2%)	
With Unilateral pleural effusions	22 (25.9%)	10 (30.3%)	
With Bilateral pleural effusions	55 (64.7%)	16 (48.5%)	
Lung compression^a^ (%)	9.4 ± 4.7	41.1 ± 16.5	<0.001*
Treatment of pneumothorax^b,c^			0.670
Observation	21 (23.8%)	6 (18.2%)	
Intraoperative thoracic drainage	44 (50.0%)	16 (48.5%)	
Postoperative thoracic drainage	23 (26.1%)	11 (33.3%)	
Spinal tumor history^b^			0.028*
Primary	59 (69.4%)	15 (45.5%)	
Recurrent	26 (30.6%)	18 (54.5%)	
Operation time^a^ (h)	6.9 ± 2.5	7.1 ± 2.7	0.725
Blood loss^a^ (ml)	1574.1 ± 1138.6	2278.8 ± 1806.2	0.043*
Surgical method^b^			0.936
En bloc resection	50 (59.5%)	20 (62.5%)	
Piecemeal resection	34 (40.5%)	12 (37.5%)	
A repair to lungs and pleura^b^			0.732
No defect	28 (32.9%)	9 (27.3%)	
Pleural defect with suture repair	23 (27.1%)	10 (30.3%)	
Pleural defect with patch repair	27 (31.8%)	12 (36.4%)	
Lungs and pleural defect with patch repair	3 (3.5%)	2 (6.1%)	
Defect cannot be repaired	4 (4.7%)	0 (0.0%)	
Tumor size^a^ (cm)	6.7 ± 2.4	6.5 ± 2.6	0.791
Surgical approach^b^			0.418
Anterior	0 (0.0%)	1 (3.0%)	
Posterior	83 (97.6%)	31 (93.9%)	
Combined	2 (2.4%)	1 (3.0%)	
Surgical segment^b^			0.126
Cervicothoracic	3 (3.5%)	2 (6.1%)	
Thoracic	77 (90.6%)	30 (90.9%)	
Thoracolumbar	4 (4.7%)	0 (0.0%)	
Lumbar	1 (1.2%)	1 (3.0%)	
Spinal levels involved^a^ (n)	1.9 ± 0.9	2.3 ± 1.1	0.143
Reconstruction^b^			0.732
Artificial vertebral body	27 (31.8%)	8 (25.0%)	
Titanium mesh	27 (31.8%)	14 (43.8%)	
Bone cement	3 (3.5%)	0 (0.0%)	
Others	5 (5.9%)	1 (3.1%)	
No reconstruction	23 (27.1%)	9 (28.1%)	
Pathology^b^			0.596
Giant cell tumor of bone	11 (12.9%)	7 (21.9%)	
Chondrosarcoma	16 (18.8%)	3 (9.4%)	
Neurogenic tumor	14 (16.5%)	5 (15.6%)	
Hemangioma	8 (9.4%)	6 (18.8%)	
Spinal metastases	13 (15.3%)	3 (9.4%)	
Myeloma	7 (8.2%)	2 (6.3%)	
Ewing Sarcoma	3 (3.5%)	2 (6.3%)	
Osteosarcoma	2 (2.4%)	2 (6.3%)	
Chordoma	2 (2.4%)	0 (0.0%)	
Others	9 (10.6%)	2 (6.3%)	

^a^ The values are given as the mean and the standard deviation.

^b^ The values are given as the number of patients, with the percentage in parentheses.

^c^ The total frequency exceeds the total number of patients because some patients had more than 1 symptoms or had bilateral pneumothorax.

* These *p*-values were less than 0.05.

## Discussion

The term “pneumothorax” was first coined by Itard in 1803, later characterized by Laennec in 1819, and denotes the collection of air in the pleural cavity. Most patients have a rapid onset and often present with sudden chest pain, tightness, and dyspnea. Nonetheless, some patients have unobvious symptoms that require imaging tests to confirm. Due to the particular characteristics of thoracic and upper lumbar tumors, the proportion of pleural rupture during surgery is higher, and the possibility of post-operative pneumothorax is greater. Therefore, pneumothorax after thoracic and lumbar tumor resection is a complication that cannot be ignored. There is no comprehensive research on the diagnosis and treatment of this frequently neglected complication.

Based on our analysis, the following reasons may contribute to the occurrence of pneumothorax after spinal tumor surgery. First, in patients with primary thoracic tumors with huge paravertebral soft tissue masses that adhere to the pleura tightly or even invade part of the pleura or the lungs, an *en bloc* resection with a wide margin is often necessary to remove the tumor together with the involved pleura and lung tissues. Then the 4-0 prolene suture is used to close the lungs and the pleura. If the defect area is too large to be sutured directly, an artificial dura mater patch will be used to repair the pleura. If the ligation line falls off after surgery, pneumothorax may occur. Second, scar tissue adhesion to the pleura is severe in spinal tumor revision surgery, and there are no normal tissue boundaries. During tumor dissection, it is easy to tear the pleura. Another situation occurred in one case of our series, in which no evident pleural breach was noticed during operation but the residual pleura became extremely thin due to wide excision of the tumor, and a delayed rupture of the pleura occurred after surgery, resulting in a pneumothorax (Figure 4). Finally, some patients have previous pulmonary diseases such as COPD, bronchiectasis, or bullae. After surgery, the transpulmonary pressure suddenly increases as a result of coughing and sputum expectoration, which may lead to destruction of the alveolar wall or bullae. Consequently, air enters the pleural cavity to form a pneumothorax. In our study, two patients with lower lumbar tumors developed pneumothorax after surgery, which might be due to this reason.

Patients with postoperative pneumothorax due to pleural rupture are often accompanied by pleural effusion and lung infection. Consequently, the lungs further collapse, which directly affects the ventilation function, aggravates hypoxia and carbon dioxide retention, and induces respiratory failure, multiple organ dysfunction, or even death. Due to the application of postoperative analgesics, the symptoms of pneumothorax in patients are not typical. Bedside, chest radiographs in the supine position cannot clearly identify pneumothorax, and some spine surgeons lack a comprehensive understanding of postoperative pneumothorax, all of which contribute to pneumothorax after spinal tumor surgery being easily missed.

Compared with spontaneous pneumothorax, postoperative pneumothorax should be treated more positively. After years of clinical practice and exploration, our center has developed an algorithmic approach to the prevention, diagnosis and management of pneumothorax following thoracic or lumbar tumor surgery. Ample evidence suggests that COPD or emphysema (15), cystic fibrosis (16), severe asthma (17), tuberculosis (18), lung cancer (19) and interstitial lung disease (20) may lead to secondary pneumothorax. In terms of preoperative prevention, for patients with spinal tumors, chest x-rays, blood gas analysis, and lung function evaluation should be performed before surgery to assess the reserve capacity of the lungs. If a patient presents severe restrictive ventilatory dysfunction, chest CT should be performed to rule out the existence of lung pathological changes. Smokers should abstain from tobacco for at least 2 weeks before spine surgery. Preoperative inspiratory muscle training is effective to reduce postoperative pulmonary complications (21).

During the surgery, Spine surgeons should not only be familiar with the anatomy of the spine and its surrounding structures but also master the surgical techniques of meticulous dissection. In the process of dissecting the soft tissue on both sides of the vertebral tumor, special attention should be paid to the separation of the adhesion between the pleura and the tumor capsule. The pleura is easily torn during the process of removing the vertebral tumor by rotating around the dural sac. To minimize the risk, we can temporarily decrease the tidal volume for a short period of time to create more surgical manipulating space for rotating the spinal tumor safely. If the pleural tear occurs during the operation, the pleura should be closed in time with a 4-0 prolene suture. If the pleural defect area is too large to be sutured directly, it can be repaired with an artificial dura mater patch. When the pleural defect cannot be sutured or repaired, a chest drainage tube should be placed on the affected side after the operation.

In terms of postoperative management, Patients need to be confined in bed for 1–2 weeks after spinal tumor surgery and cannot undergo a CXR in a standing position. On a supine chest radiograph, the only sign of pneumothorax may be a deep lateral costophrenic angle on the involved side, which is called the deep sulcus sign (22). Besides, patients with intraoperative pleural tears are often complicated by pneumonia and pleural effusion after surgery, which decreases the sensitivity of CXR in diagnosing postoperative pneumothorax. Therefore, if the patient's vital signs are stable, we recommend chest CT as the preferred examination for pneumothorax after spinal tumor surgery. CT scan can not only identify small pneumothoraces but also give a precise assessment of the pneumothorax size. Thoracic sonography has been increasingly used in the diagnosis of pneumothorax. A meta-analysis of 13 studies (23), demonstrated that thoracic ultrasonography offered a 78% sensitivity and a 98% specificity vs. 39% and 99% for CXR. However, the diagnostic reliability of ultrasonography is highly dependent on the examiner's experience and it is impossible to assess the size of a pneumothorax. Therefore, ultrasonography should be supplemented with a supine CXR to rule out pneumothorax, particularly in patients who are unable to be transferred for chest CT.

Postoperatively, it is very important to teach patients about inspiratory muscle training in the early stages (24). We also recommend instructing patients about effective and appropriate expectoration to avoid persistent and severe coughing. We suggest that patients with small post-operative pneumothorax without dyspnea should be observed closely. Clinical and imaging evaluations including chest CT or bedside CXR should be performed again after 24 h. If small pneumothorax develops into large pneumothorax or dyspnea aggravates progressively, closed chest drainage should be performed in time. Tension pneumothorax is a potentially life-threatening event that needs immediate intervention for the existing or expected cardiovascular depression or progressive dyspnea. In the case of large or symptomatic pneumothorax, we recommend the application of a closed chest drain. Re-expansion edema may develop in very few patients following chest drainage. In very rare cases, intubation is required due to the dissemination of pulmonary edema to both lungs (25). Patients with re-expansion edema should be identified early and dealt with promptly to avoid poor prognosis. The indication for the removal of the chest tube is adequate re-expansion of both lungs and the absence of air leakage. We recommend surgical therapy in patients with persistent air leaks or insufficient re-expansion after chest drain therapy.

The limitation of this study is its single-center retrospective design. Potential selection bias and confounding bias are inevitable. In this study, we mainly described the clinical features of pneumothorax in patients receiving thoracic and lumbar tumor surgery and discussed its diagnosis and intervention. Further investigation is required to clarify the underlying risk factors leading to pneumothorax after spinal tumor surgery. In addition, the overall incidence of postoperative pneumothorax is also difficult to estimate, because not all patients with thoracic and lumbar tumors underwent chest CT or chest x-ray postoperatively. Thus, some asymptomatic patients with pneumothorax would be missed. Prospective, multicenter cohort studies are needed to determine the overall incidence of postoperative pneumothorax.

## Conclusions

In conclusion, most patients with pneumothorax following thoracic and lumbar tumor surgery had no history of smoking or pre-existing pulmonary diseases. Spinal tumor history and intraoperative blood loss were risk factors for large pneumothorax. During the surgery, an artificial dura mater patch and prolene suture can be used to repair the pleural and lung defects. If the patient's pleural defect cannot be sutured or repaired, placement of a chest drainage tube during the surgery can help reduce the degree of postoperative pneumothorax or pleural effusion. Chest CT should be the preferred examination due to its high sensitivity. We recommend observation in small pneumothorax patients without dyspnea and pleural effusion. If the patient develops severe dyspnea, large pneumothorax, or concurrent pleural effusion, application of chest drainage is indicated.

## Data Availability

The raw data supporting the conclusions of this article will be made available by the authors, without undue reservation.
